# Global Cicada Sound Collection I: Recordings from South Africa and Malawi by B. W. Price & M. H. Villet and harvesting of BioAcoustica data by GBIF

**DOI:** 10.3897/BDJ.3.e5792

**Published:** 2015-09-02

**Authors:** Ed Baker, Benjamin Wills Price, Simon Rycroft, Martin H. Villet

**Affiliations:** ‡ The Natural History Museum, London, United Kingdom; § Rhodes University, Grahamstown, South Africa

**Keywords:** bioacoustics, BioAcoustica, Cicadidae, acoustic recording, Malawi, South Africa, GBIF

## Abstract

**Background:**

Sound collections for singing insects provide important repositories that underpin existing research (e.g. Price et al. 2007 at http://bio.acousti.ca/node/11801; Price et al. 2010) and make bioacoustic collections available for future work, including insect communication (Ordish 1992), systematics (e.g. David et al. 2003), and automated identification (Bennett et al. 2015). The BioAcoustica platform (Baker et al. 2015) is both a repository and analysis platform for bioacoustic collections: allowing collections to be available in perpetuity, and also facilitating complex analyses using the BioVeL cloud infrastructure (Vicario et al. 2011). The Global Cicada Sound Collection is a project to make recordings of the world's cicadas (Hemiptera: Cicadidae) available using open licences to maximise their potential for study and reuse. This first component of the Global Cicada Sound Collection comprises recordings made between 2006 and 2008 of Cicadidae in South Africa and Malawi.

**New information:**

This collection of sounds includes 219 recordings of 133 voucher specimens, comprising 42 taxa (25 identified to species, all identified to genus) from South Africa and Malawi. The recordings have been used to underpin work on the species limits of cicadas in southern Africa, including Price et al. (2007) and Price et al. (2010). The specimens are deposited in the Albany Museum, Grahamstown, South Africa (AMGS).

The harvesting of acoustic data as occurrence records by GBIF has been implemented by the Scratchpads Team at the Natural History Museum, London. This link increases the value of individual recordings and the BioAcoustica platform within the global infrastructure of biodiversity informatics by making specimen/occurence records from BioAcoustica available to a wider audience, and allowing their integration with other occurence datasets that also contribute to GBIF.

## Introduction

BioAcoustica (Baker et al. 2015) is an online database and analysis platform for recorded wildlife sound and is based on the Scratchpads (Smith et al. 2011) virtual research environment.

Sound collections which include some Cicada recordings include the Macaulay Library, Cornell Lab of Ornithology (http://macaulaylibrary.org/: 148 recordings), the Animal Sound Archive of the Museum für Naturkunde in Berlin (http://www.animalsoundarchive.org/: 11 recordings) and Wikimedia Commons (https://commons.wikimedia.org/wiki/Category:Audio_files_of_Cicadidae: 13 recordings). In addition while region specific cicada sound collections do exist (Table 1), the aim of this resource is to provide a mechanism by which distributed collections of recordings can be made available in human and machine readable formats. The collection described in this paper with 219 recordings is the second largest collection of cicada songs made available.

## General description

### Purpose

We have used the BioAcoustica platform to start creating an online, freely accessible, openly licensed and global resource for anybody interested in the bioacoustics of cicadas: the Global Cicada Sound Collection (GCSC). The collection of recordings made by Price, Villet and collaborators of southern African Cicadidae is the first collection to be made available through the GCSC project. We are currently working with other collaborators internationally to make their collections available. As the GCSC will include multiple collections, made available over a long time period, individual collaborators will be publishing data papers on their contributions as they are made available (if they choose to do so). This method allows for the entire collection to be made available for research while preserving the credit of contributors through granular citation of contributions.

### Additional information

The use of the BioAcoustica platform allows for recordings to be shared with the Encyclopedia of Life (Parr et al. 2014) using a DarwinCore Archive (Baker et al. 2014). BioAcoustica metadata is archived at the Natural History Museum's Data Portal (Baker et al. 2014a).

## Project description

### Title

Digitising Southern African Cicada Sounds for the Global Cicada Sound Collection

### Personnel

Field recordings were made by Benjamin W. Price, M. H. Villet and others between 2006 and 2008. The collection was prepared for online availability by Ed Baker.

### Funding

Funding for making the recordings available online was obtained by Price, Baker & Vincent S. Smith as part of the Natural History Museum Departmental Investment Fund (DIF) award SDF 14011. The recordings were made on fieldwork funded by Rhodes University (grant number 37201) and the National Research Foundation (NRF) of South Africa to Villet (grant number 65774) and Price (grant number 67389). Any opinion, findings and conclusions or recommendations expressed in this material are those of the author and do not necessarily reflect the views of the NRF.

## Geographic coverage

### Description

This collection of sound recordings includes cicada sounds from across South Africa and Malawi.

### Coordinates

-34.75 and -10.817 Latitude; 17.602 and 34.264 Longitude.

## Taxonomic coverage

### Description

The taxon list includes only those taxa identified to species present in the collection.

### Taxa included

**Table:** 

Rank	Scientific Name	Common Name
species	*Azanicada zuluensis*	
species	*Brevisiana brevis*	
species	*Ioba leopardina*	
species	*Munza furva*	
species	*Munza laticlavia*	
species	*Orapa numa*	
species	*Oxypleura lenihani*	
species	*Platypleura argentata*	
species	*Platypleura brunea*	
species	*Platypleura capensis*	
species	*Platypleura chalybaea*	
species	*Platypleura deusta*	
species	*Platypleura divisa*	
species	*Platypleura haglundi*	
species	*Platypleura hirta*	
species	*Platypleura hirtipennis*	
species	*Platypleura maytenophila*	
species	*Platypleura mijburghi*	
species	*Platypleura plumosa*	
species	*Platypleura signifera*	
species	*Platypleura stridula*	
species	*Platypleura techowi*	
species	*Platypleura wahlbergi*	
species	*Pycna semiclara*	
species	*Pycna sylvia*	

## Temporal coverage

**Living time period:** 2006-2008.

## Collection data

### Collection name

Albany Museum

### Collection identifier

AMGS

### Specimen preservation method

Pinned or 70% Ethanol

### Curatorial unit

Species collecting event

## Usage rights

### Use license

Other

### IP rights notes

Recordings and metadata are released under a Creative Commons Attribution (CC-BY) licence. BioAcoustica has a fine-grained licensing mechanism, where recordings are individually licenced. Other projects may have alternative licences. Copyright of the recordings belongs to the individual sound recordists.

## Data resources

### Data package title

Global Cicada Sounds Collection

### Number of data sets

1

### Data set 1.

#### Data set name

GCSC 1: South Africa and Malawi

#### Number of columns

3

#### Download URL

http://bio.acousti.ca/project/620

#### Description

This resource is a summary of the recordings included in this project. The full BioAcoustica dataset, including this and other projects, is available in DarwinCore Archive format (as described in Baker et al. (2014)) at http://bio.acousti.ca/dwca.zip or from Baker et al. (2014a).

**Data set 1.** 

Column label	Column description
Recording	HTML link to BioAcoustica recording page
Specimen	HTML link to BioAcoustica specimen/observation page
Location	HTML link to BioAcoustica location page associated with Specimen

## Additional information

### Harvesting of occurrence records by the Global Biodiversity Informatics Facility (GBIF)

Each Scratchpad automatically registers itself with the GBIF registry as a dataset. These datasets are associated with two entities within the registry, an organization, and an installation. The organization is 'Scratchpads', and the installation is 'Scratchpads at Natural History Museum, London', which theoretically allows additional Scratchpad installations at different institutions. The Scratchpads organization is sponsored by the UK's National Biodiversity Network, a requirement of the GBIF infrastructure.

On top of this, if a Scratchpad has a web service capable of providing data, then it is added as an endpoint to the dataset. We use a Darwin Core Archive (DwC-A) file as an endpoint to provide GBIF with the data from the sounds database.

The BioAcoustica dataset can be accessed at: http://www.gbif.org/dataset/30f55c63-a829-4cb2-9676-3b1b6f981567.

## Figures and Tables

**Table 1.** Current online Cicada specific acoustic repositories.

Geographic extent	Title	Link	Recordings
North America	Insect Singers	http://www.insectsingers.com/	112
North America	Cicada Mania	http://www.cicadamania.com/audio/	60
Michigan	Cicadas of Michigan	http://insects.ummz.lsa.umich.edu/fauna/Michigan_Cicadas/Michigan/Index.html	15
South East Asia	Phantastic songs of the S.E.Asian cicadas!	http://www2.arnes.si/~ljprirodm3/asian_cicadas.html	7
Japan and Korea	Cicadidae in Japan	http://homepage2.nifty.com/saisho/cicadasongaac_e.html	148
Borneo	Cicada songs from Borneo	http://www.groms.de/data/zoology/riede/cicada.html	5
Europe	Songs of European Singing Cicadas	http://www.cicadasong.eu/	74
Slovenia, Croatia and Macedonia	Songs of Cicadas from Slovenia, Croatia and Macedonia	http://www2.arnes.si/~ljprirodm3/cikade.html	12
Australia	A web guide to the cicadas of Australia	http://dr-pop.net/cicadas.htm	351
